# Predicting current and future habitat of Indian pangolin (*Manis crassicaudata*) under climate change

**DOI:** 10.1038/s41598-024-58173-w

**Published:** 2024-03-30

**Authors:** Siddiqa Qasim, Tariq Mahmood, Bushra Allah Rakha, Muhammad Sajid Nadeem, Faraz Akrim, Asad Aslam, Jerrold L. Belant

**Affiliations:** 1 Department of Zoology, Wildlife and Fisheries, PMAS Arid Agriculture University, Rawalpindi, Pakistan; 2 Department of Zoology, University of Kotli, Azad Jammu and Kashmir, Pakistan; 3 Department of Fisheries and Wildlife, Michigan State University, East Lansing, MI USA

**Keywords:** Climate-change ecology, Conservation biology, Ecological modelling

## Abstract

Climate change is among the greatest drivers of biodiversity loss, threatening up to 15–30% of described species by the end of the twenty-first century. We estimated the current suitable habitat and forecasted future distribution ranges of Indian pangolin (*Manis crassicaudata*) under climate change scenarios. We collected occurrence records of Indian pangolin using burrow counts, remote camera records and previously published literature in Pakistan during 2021–2023. We downloaded bioclimatic data for current (1970–2000) and future (2041–2060, 2061–2080, 2081–2100) climate scenarios from the WorldClim database using the Hadley Global Environment Model (HadGEM3-GC31-LL). We used MaxEnt software to predict current and future distributions of Indian pangolin, then computed the amount of habitat lost, gained, and unchanged across periods. We obtained 560 Indian pangolin occurrences overall, 175 during the study, and 385 from our literature search. Model accuracy was very good (AUC = 0.885, TSS = 0.695), and jackknife tests of variable importance showed that the contribution of annual mean temperature (bio1) was greatest (33.4%), followed by the annual precipitation (bio-12, 29.3%), temperature seasonality (bio 4, 25.9%), and precipitation seasonality (bio 15, 11.5%). The maxent model predicted that during the current time period (1970–2000) highly suitable habitat for Indian pangolin was (7270 km^2^, 2.2%), followed by moderately suitable (12,418 km^2^, 3.7%), less suitable (49,846 km^2^, 14.8%), and unsuitable habitat (268,355 km^2^, 79.4%). Highly suitable habitat decreased in the western part of the study area under most SSPs and in the central parts it declined under all SSPs and in future time periods. The predicted loss in the suitable habitat of the Indian pangolin was greatest (26.97%) under SSP 585 followed by SSP 126 (23.67%) during the time 2061–2080. The gain in suitable habitat of Indian pangolin was less than that of losses on average which ranged between 1.91 and 13.11% under all SSPs during all time periods. While the stable habitat of the Indian pangolin ranged between 64.60 and 83.85% under all SSPs during all time periods. Our study provides the current and future habitat ranges of Indian pangolin in the face of a changing climate. The findings of our study could be helpful for policymakers to set up conservation strategies for Indian pangolin in Pakistan.

## Introduction

Biodiversity is under many anthropogenic threats globally, including habitat degradation, habitat loss, biological invasions, overexploitation, pollution, and climate change^1–5^. It is expected that due to climate change, up to 15–30% of described species will be threatened as rising temperatures and weather patterns influence the physiological tolerances of many species^6–8^. Variations in temperature and precipitation patterns due to climate change alter species distributions that may lead to population declines, extinctions, range shifts, range losses, disease transmission, and abrupt trophic interactions^9–11^. Climate change in Anthropocene is largely driven by anthropogenic activity and the rate of future climate change will depend on the growth of human population, resource and land use, and mitigation strategies. Species extinctions and the factors causing them to vary regionally^12–14^. Mammal species with low population densities and reproductive rates are more susceptible to anthropogenic threats^15–17^.

Knowledge of species' geographic distributions and factors affecting these distributions are fundamental for conservation planning, and forecasting future actions^18^, and can play an important role in ecological restoration^19–21^. This information is also needed to understand the ecological and evolutionary determinants of biodiversity distribution patterns^22^.

Species Distribution Models (SDMs) are used to estimate species’ habitat, environment relationships, and predict current and future distributions. These models are often used in the ecology and conservation of species and their estimated responses to current and future climatic conditions^23,24^. Species distribution models have been used to assist the direction of field surveys, assess the effects of climate change, and improve conservation planning^25–28^.

Ecological knowledge of the species' potential distribution and suitable habitats, facing sharp population decline across its distribution range is crucial for long-term conservation planning^29^. For Manis species, the occurrence is determined mainly by food availability (i.e., presence of ants and termites), burrows (living and feeding), suitable temperatures, and the presence of water sources^30–34^. In addition, distance to human settlements or roads can also influence pangolin occurrence^24,29,35^.

The Indian pangolin (*Manis crassicaudata*) is categorized as endangered by the International Union for Conservation of Nature (IUCN) Red List of Threatened Species^36^ and listed in ESM Appendix I of the Convention on International Trade in Endangered Species of Wild Fauna and Flora^37^. The Indian Pangolin faces population declines from illegal killing due to high demand for its scales^31,38,39^ in medicinal^40^ wrong myths^29^ and ornamental use^41^.

The distribution of Indian pangolin is influenced by temperature, precipitation, elevation, ants and termites, human settlements, landcover, and other factors^24,29,42–46^. Indian pangolins prefer sites with moderate canopy cover and slope, not far from human settlements and water, at moderate elevation (500–1750 m above sea level)^42,46^. Indian pangolin occupies diverse habitats including thorn forests, pine forests, agricultural lands, plains, and grasslands^47^.

There are no estimates of habitat distribution for Indian pangolins in Azad Jammu and Kashmir (AJ&K) and north Pakistan. Our objective was to estimate the distribution of pangolin habitat in AJ&K and north Pakistan and forecast habitat under accepted climate change scenarios. We predicted that the habitat suitability of the Indian pangolin will be negatively influenced by increasing temperature while it will be positively influenced by rainfall. We further predicted that the suitable habitat of the Indian pangolin will shrink under climate change scenarios.

## Results

**Figure 1:**
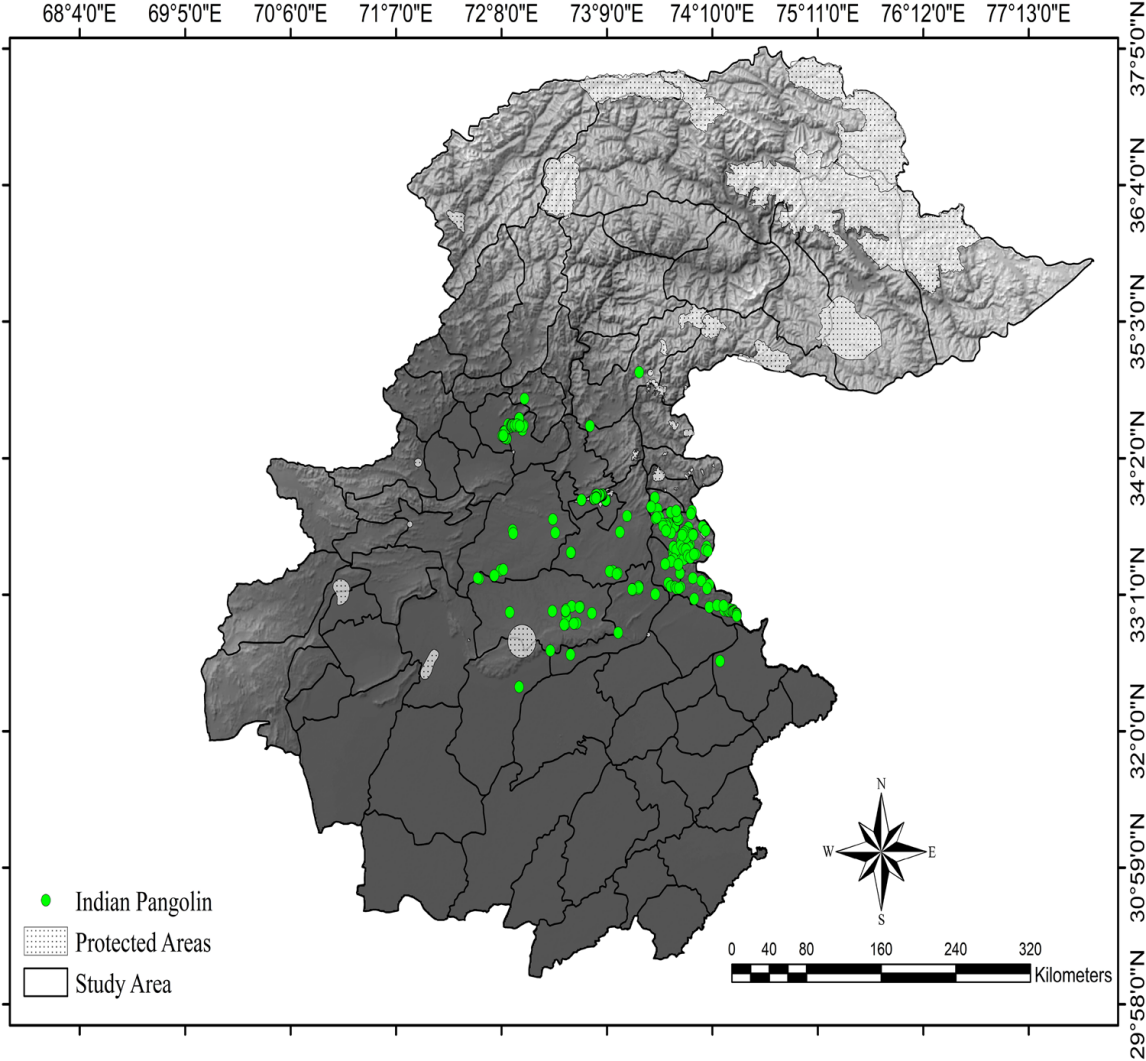
Study area with occurrence points of Indian pangolin, northern Pakistan, 2021–2023. Created using Arc GIS (version 10.3, https://www.esri.com/arcgis-blog/products/3d-gis/3d-gis/arcgis-10-3-the-next-generation-of-gis-is-here/).

We obtained 560 Indian pangolin occurrences overall, 175 during the study, and 385 from our literature search (Fig. 1). Spatial filtering yielded 159 occurrences for modeling.

**Figure 2:**
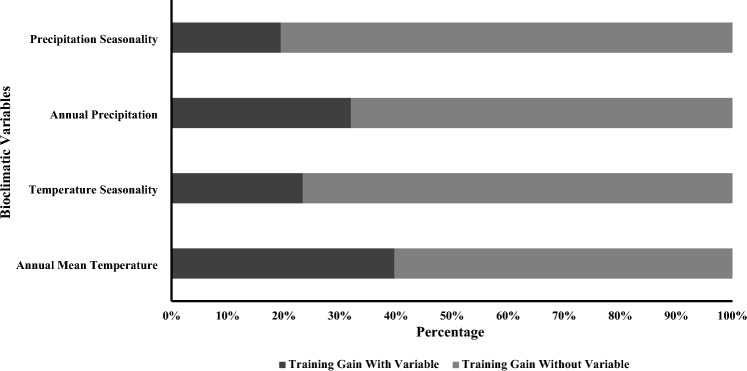
Jackknife of regularized training gain for Indian pangolin showing contribution of bioclimatic variables to the Maxent model, northern Pakistan, 2021–2023.

Of the initial 19 bioclimatic variables, we removed 15 variables that were highly correlated (*r* > 0.7) and retained 4 variables for our models. Model accuracy was very good (AUC = 0.885, TSS = 0.695), and jackknife tests of variable importance showed that the contribution of annual mean temperature (bio1) was greatest (33.4%), followed by mean temperature of coldest quarter (bio 12, 29.3%), temperature seasonality (bio 4, 25.9%), and precipitation seasonality (bio 15, 11.5%) (Table 1, Fig. 2).

**Table 1:** Current and future habitat suitability of Indian pangolin under Shared Socioeconomic Pathways (SSPs) using Global Climate Model (GCM) Hadley global environment (HadGEM3-GC31-LL).

Time period	Scenario	Highly suitable (%)	Moderately suitable (%)	Less suitable (%)	Not suitable (%)
1970–2000	Current	7269.97 (2.15)	12,417.63 (3.68)	49,846.48 (14.75)	268,354.77 (79.42)
2041–2060	SSP126	6107.97 (1.81)	12,075.67 (3.58)	44,511.24 (13.21)	274,280.14 (81.39)
	SSP 245	7222 (1.78)	16,189 (3.99)	53,729 (13.23)	328,931 (81)
	SSP 585	6677.35 (1.98)	14,789.77 (4.39)	44,024.86 (13.06)	271,578.49 (80.57)
2061–2080	SSP126	6042.4 (1.79)	23,368.65 (6.93)	32,325.18 (9.59)	275,319.3 (81.68)
	SSP 245	6380.21 (1.89)	18,745.55 (5.56)	32,540.15 (9.65)	279,469.3 (82.9)
	SSP 585	6102.99 (1.81)	24,401.17 (7.24)	29,997.86 (8.9)	276,700.42 (82.06)
2081–2100	SSP126	5194.14 (1.54)	9839.65 (2.92)	36,764.85 (10.91)	285,312.5 (84.63)
	SSP 245	7389.49 (2.19)	19,293.35 (5.71)	32,750.14 (9.69)	278,455.04 (82.41)
	SSP 585	6132.04 (1.82)	15,197.3 (4.52)	38,977.63 (11.58)	276,288.74 (82.08)

**Figure 3:**
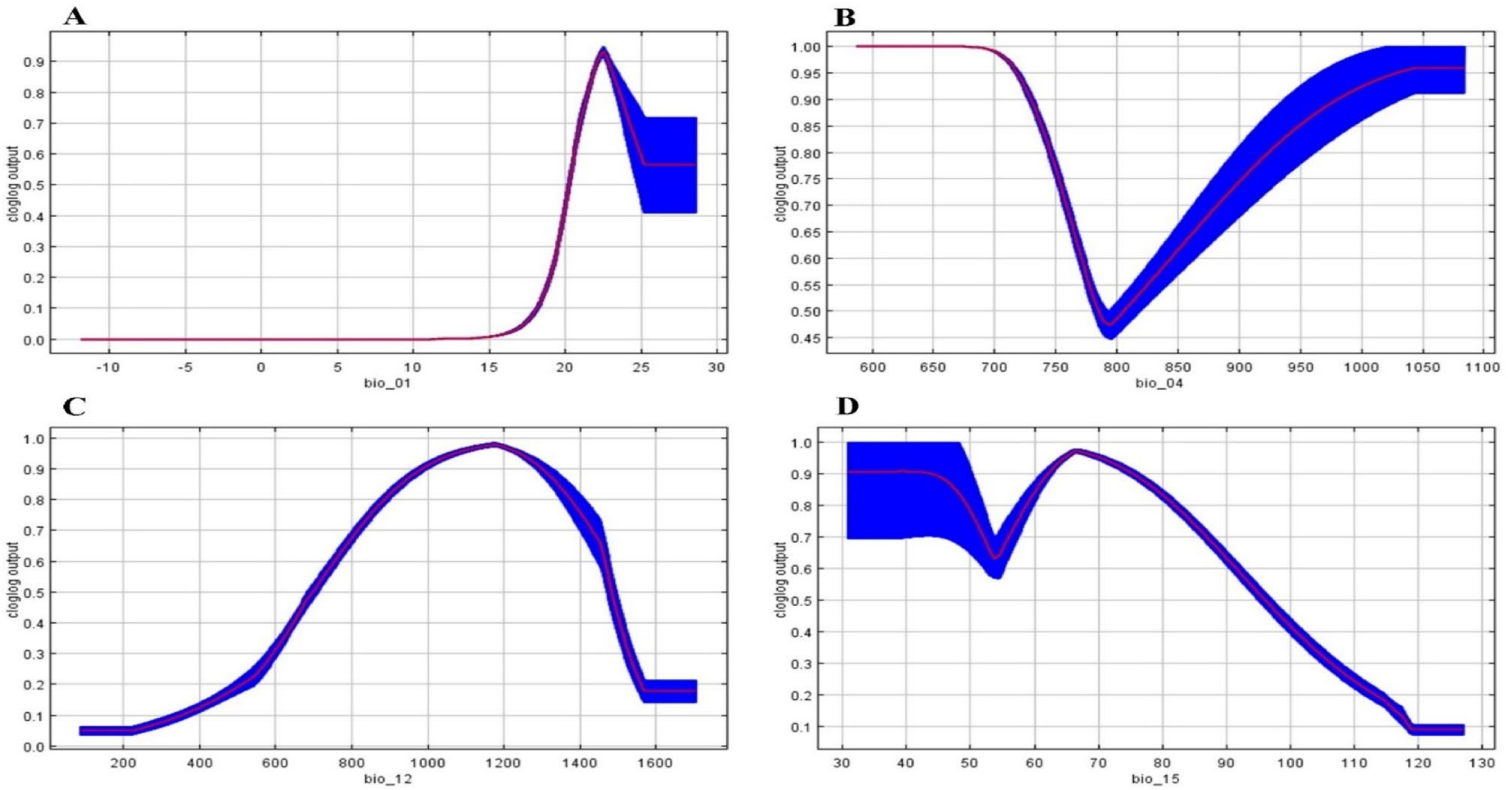
Response of bioclimatic variable to habitat suitability of Indian pangolin (red line represents standard deviations, blue line represents effects of bioclimatic variables on predicted habitat suitability), northern Pakistan, 2021–2023. (**A**) Annual Mean Temperature (Bio1), (**B**) Temperature Seasonality (Bio 04), (**C**) annual precipitation (Bio12), **D**) Precipitation Seasonality (Bio 15).

Habitat suitability for Indian pangolins increased to 0.94 with annual mean temperature increasing from 15 to 22 °C, then declined to 0.58 when temperature increased to 28 °C. The habitat suitability of the Indian pangolin decreased (1–0.48) with increasing temperature seasonality and habitat suitability increased to 0.96 when temperature seasonality was above 1050. The habitat suitability for Indian pangolin increased with increasing annual precipitation (bio12) and peaked (0.98) at 1100–1200 values. The habitat suitability of the Indian pangolin decreased with increasing precipitation seasonality (bio15, Fig. 3).

**Figure 4:**
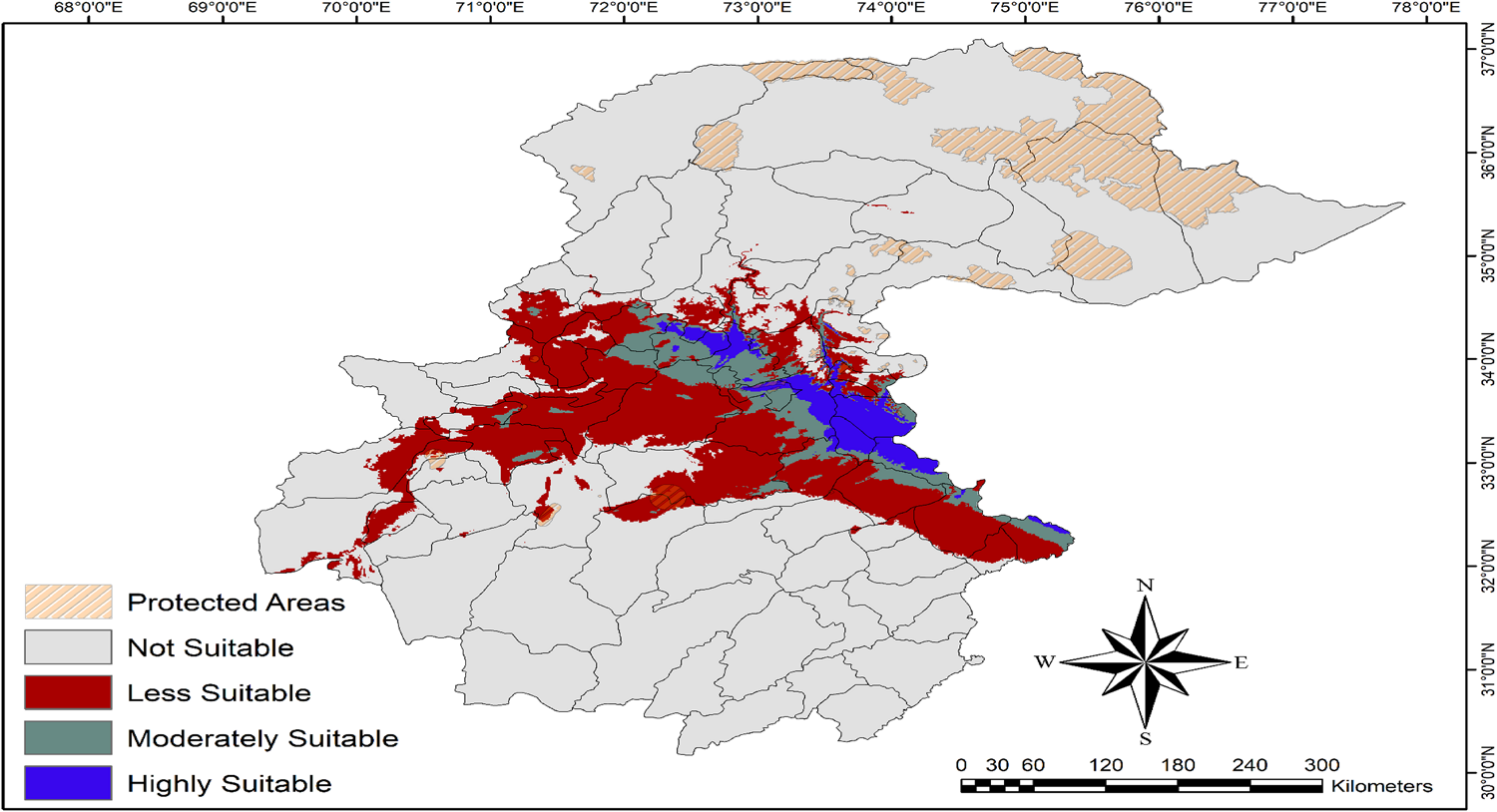
Current habitat suitability of Indian pangolin, northern Pakistan. Created using Arc GIS (Version 10.3, https://www.esri.com/arcgis-blog/products/3d-gis/3d-gis/arcgis-10-3-the-next-generation-of-gis-is-here/).

The maxent model predicted that highly suitable habitat for Indian pangolins was (7269.97 km^2^, 2.15%), followed by moderately suitable (12,417.63 km^2^, 3.68%), less suitable (49,846.48 km^2^, 14.75%), and unsuitable habitat (268,354.77 km^2^, 79.42%) (Table 1, Fig. 4). The most highly suitable habitat for the Indian pangolin was in the eastern, central, and western parts of the study area. The moderately suitable habitat was mainly in the central followed by the western and eastern parts of the study area.

**Figure 5:**
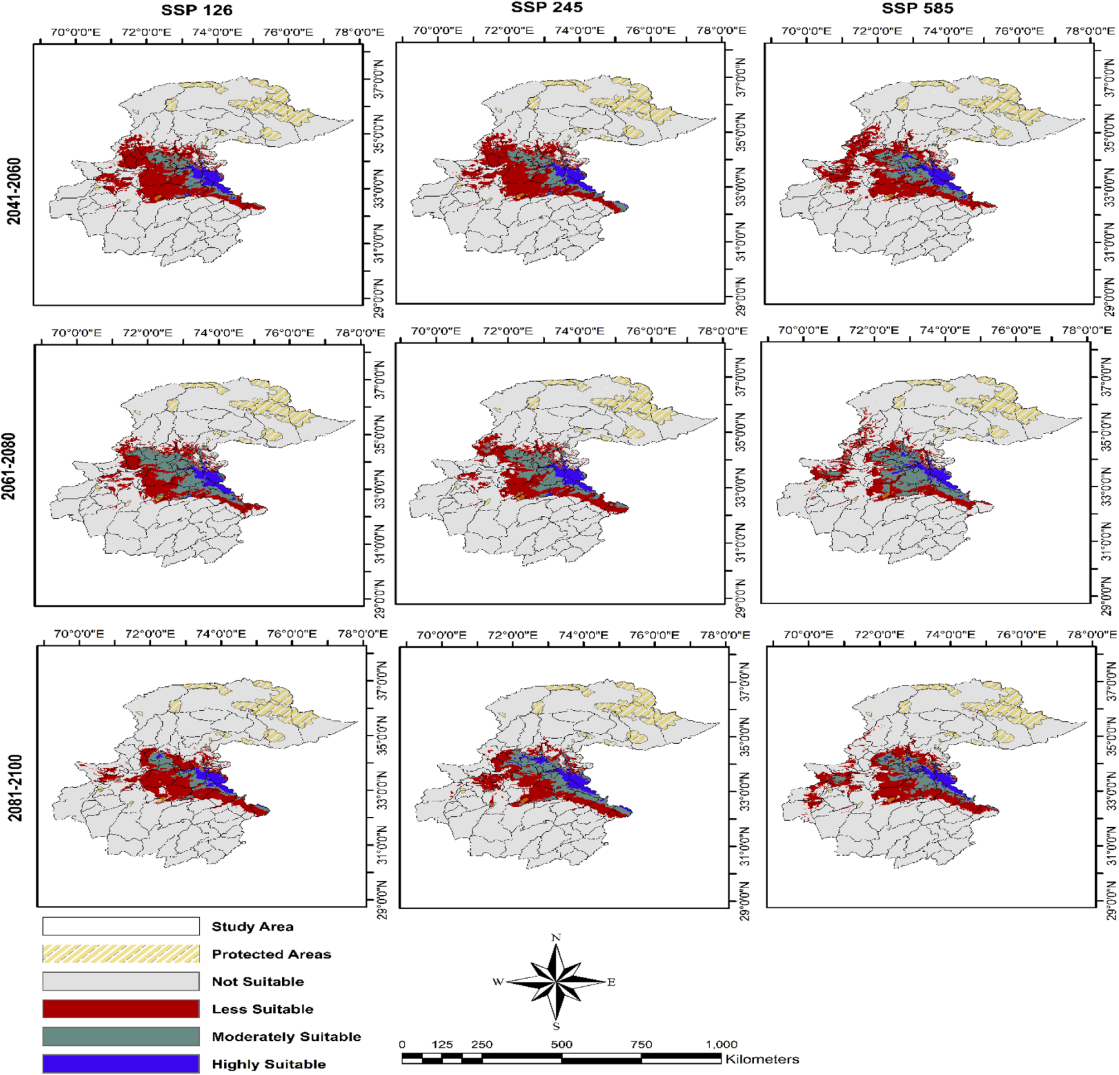
Future Habitat Suitability of Indian pangolin in northern Pakistan under different climate change scenarios under SSP126, SSP 245, SSP585 and during time periods 2041–2060, 2061–2080, 2081–2100. Created using Arc GIS (Version 10.3, https://www.esri.com/arcgis-blog/products/3d-gis/3d-gis/arcgis-10-3-the-next-generation-of-gis-is-here/).

The predicted highly suitable habitat for Indian pangolin during 2041–2060 under three SSPs was (1.78–1.98%), moderately suitable was 3.58–4.39%, less suitable was 13.06–13.23%, and unsuitable was 80.57–81.39%. The predicted highly suitable during 2061–2080 under three SSPs for Indian pangolin was 1.79–1.89%, moderately suitable 5.56–7.24%, less suitable was 8.9%-9.65% and not suitable was 81.68–82.9%. The predicted highly suitable habitat during 2081–2100 under three SSPs was 1.54–2.19%, moderately suitable was 2.92–5.71%, less suitable was 9.69–11.58%, and not suitable was 82.08–84.63% (Table 1, Fig. 5). Our model showed that the highly suitable habitat of Indian pangolins in the future diminished from the western part of the study area under most SSPs and time periods except for SSP 585 (2041–2060), SSP 585 (2061–2080), and three SSPs during 2081–2100. The highly suitable habitat from central parts of the study areas also declined under all SSPs and time periods in the future. The major proportion of highly suitable habitats for Indian pangolin in the future was represented in the eastern part of the study area.

The predicted loss in the suitable habitat of the Indian pangolin was greatest (26.97%) under SSP 585 followed by SSP 126 (23.67%) during the time 2061–2080. While habitat loss under all SSPs during 2041–2060 was 9.77–12.13% and during 2081–2100 was 11.08–15.33%.

**Figure 6:**
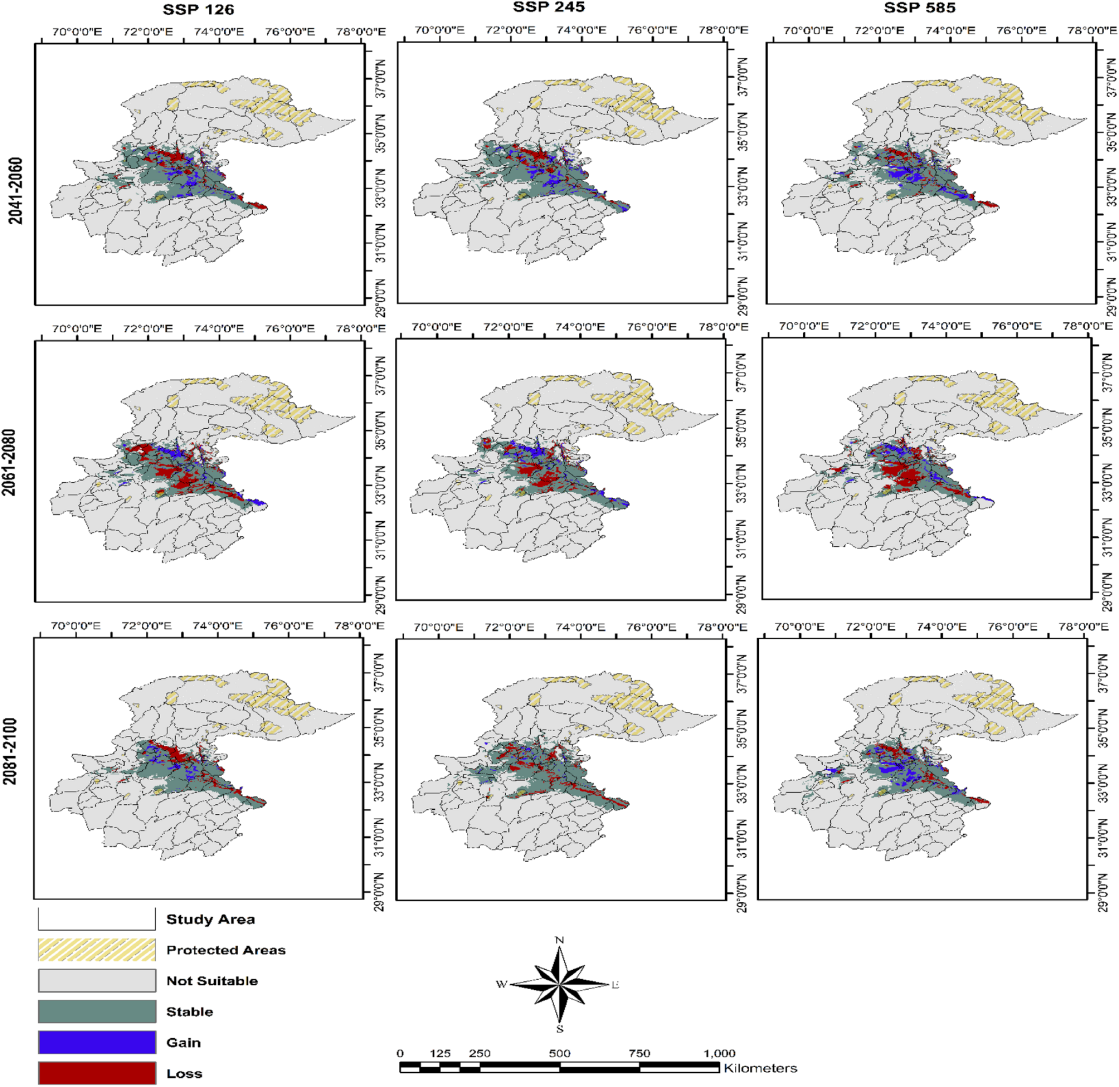
Change Analysis Stable, gain and loss in suitable habitat of Indian pangolin in northern Pakistan under different climate change scenarios under SSP126, SSP 245, SSP585 and during time periods 2041–2060, 2061–2080, 2081–2100. Created using Arc GIS (Version 10.3, https://www.esri.com/arcgis-blog/products/3d-gis/3d-gis/arcgis-10-3-the-next-generation-of-gis-is-here/).

The gain in suitable habitat of Indian pangolin was less than that of losses on average which ranged between 1.91 and 13.11% under all SSPs during all time periods. While the stable habitat of the Indian pangolin ranged from 64.60 to 83.85% under all SSPs during all time periods. Most of the loss in suitable habitat was in the western and central parts while the least loss was in the eastern part of the study area. Under all SSPs, during 2041–2060 the gain in habitat was mostly in central parts while under all SSPs of 2061–2080, the gain in habitat was mostly in the western part of the study area (Table 2, Fig. 6).

**Table 2:** Stable, gain, and loss in suitable habitat (km^2^) of Indian pangolin under shared socioeconomic pathways (SSPs) using Global Climate Model (GCM) Hadley global environment (HadGEM3-GC31-LL), northern Pakistan.

SSPs	Time period								
	2041–2060			2061–2080			2081–2100		
	SSP126	SSP 245	SSP 585	SSP126	SSP 245	SSP 585	SSP126	SSP 245	SSP 585
Stable (%)	42,838.79 (79.46)	43,664.64 (79.57)	39,390.14 (77.70)	44,916 (68.79)	43,490 (71.72)	36,841 (64.60)	36,926.7 (78.8)	48,415.56 (83.85)	37,290.24 (75.81)
Gain (%)	4536.78 (8.41)	5146.83 (9.38)	6353.65 (12.53)	4926 (7.54)	5795 (9.56)	4809 (8.43)	2748.96 (5.87)	1102.24 (1.91)	6449.1 (13.11)
Loss (%)	6539.57 (12.13)	6067.3 (11.06)	4952.61 (9.77)	15,454 (23.67)	11,351 (18.72)	15,380 (26.97)	7186.14 (15.33)	8224.47 (14.24)	5451.44 (11.08)

## Discussion

Our results demonstrated that four bioclimatic variables including annual mean temperature (bio1), annual precipitation (bio 12), temperature seasonality (bio4), and precipitation seasonality (bio15) were most important in describing Indian pangolin occurrence. Among these, annual mean temperature (bio1) had the greatest contribution in describing the distribution of Indian pangolin, followed by annual precipitation (bio 12), temperature seasonality, and precipitation seasonality contributed the least to the maxent model.

Our findings demonstrate that suitable habitat for Indian pangolin occurs in areas with annual average temperature of 15–22 °C and represents the optimal range for Indian pangolin occurrence in the study area. Our findings align with previous studies conducted in Pakistan, which reported that Indian pangolins are likely to occur in areas with moderate temperatures ranging from 15 to 30 °C^33^.

Habitat suitability of Indian pangolins decreased with increasing temperature seasonality and precipitation seasonality representing that the abrupt fluctuations in temperature and precipitation patterns during different seasons are not suitable for the species. Indian pangolin is an insectivore which feeds on ants and termites. Prey species of Indian pangolin such as termites require moist conditions to survive and reproduce and an increase in temperature and precipitation seasonality results in a decline in their population thereby negatively impacting Indian pangolin. With increased precipitation seasonality, suitable habitat for pangolins decreases while decreased precipitation seasonality increases suitable habitat for pangolins as termites are abundant in the wet season as compared to the dry season^48^. There are no existing studies on the impact of climate change on Indian pangolin in its distribution range however a few studies have assessed habitat suitability of Indian pangolin^24^ such as the Potohar region of Pakistan or the giant pangolin (*Smutsia gigantea)* in central Cameroon^49^. However, our findings are not comparable with these studies since they did not use bioclimatic variables to predict habitat suitability, nor did they predict suitable habitat of either species under changing climatic conditions.

The maxent model predicted only 2.2% of the study area was highly suitable and only 3.7% was moderately suitable for the occurrence of Indian pangolins. Most highly suitable habitat for Indian pangolins was in the eastern part of the study area (including the state of Azad Jammu and Kashmir; AJ&K), followed by central (Punjab) and western parts (KPK province of Pakistan) having suitable climatic conditions for this species occurrence. In the eastern part of the study area viz., AJ&K highly, suitable areas included regions of districts Bhimber, Mirpur, Kotli, Sudhnoti, Poonch, Bagh, Muzaffarabad which have moderate temperatures. While in the central part of the study area (Punjab province), some regions were highly suitable including Sialkot, Narowal, Rawalpindi, and Jhelum districts. In the western part of the study area (KPK province), Haripur, Swabi, Mardan and Buner districts had highly suitable habitat for Indian pangolin. The Margallah Hills National Park, Islamabad, was also a highly suitable area for Indian pangolin. The less suitable habitat was 14% while the vast majority of the study area 79% was not suitable for Indian pangolin. This is because of the reason that the climatic conditions in a major proportion of habitat are not in the range of optimum of Indian pangolin. Also, climatic conditions can alter the distribution and abundance of its prey species thereby impacting the habitat suitability of pangolins. Our findings are aligned with previous studies which also reported that the eastern and central parts of the study area (AJ&K and Potohar region of Punjab) contain suitable habitats for Indian pangolins^24,29–33^. Mahmood et al.^50,51^ reported the distribution of Indian pangolins from the Mansehra and Kohat districts of KPK. The Margallah Hills National Park has also been reported as suitable habitat for Indian pangolins^31,42,52^. Previous studies also reported that the distribution range of Indian pangolins has been reduced in the Potohar region due to illegal killing for their scales and cultural beliefs^24^. The current and future suitable habitat ranges of Indian pangolin are not represented by the protected area network in Pakistan. Establishing more protected areas in suitable habitat ranges for Indian pangolins could help in their conservation in Pakistan in the face of changing climate.

Climate change models are a primary source for scientists to predict future species distribution ranges. Our model predicted that future suitable habitat of the Indian pangolin will decline under the impacts of climate change in northern Pakistan. The highly suitable habitat of Indian pangolins in KPK province will diminish in the future following predictions of most of the models. Similarly, highly suitable habitats will also decline in Punjab province. However, the major proportion of current highly suitable habitat for Indian pangolin in AJ&K will remain suitable under future climate change. The state of AJ&K and many parts of Punjab could serve as a stronghold for the conservation of Indian pangolin in the face of changing climate.

Our findings suggest that Indian pangolin habitat will decrease under climate change with greatest losses (23.67–26.97%) in suitable habitats occurring during 2061–2070 under SSP 585 and SSP 126. While predicted loss in suitable habitat under all other time periods ranged from 10 to 15% of total area. The pangolin is the most illegally trafficked mammalian species worldwide due to demand for its scales in traditional Chinese medicines^31,32,53–55^. A pangolin is poached from the wild every five minutes and more than one million pangolins have been poached and illegally traded internationally in the past decade^32^. The Indian pangolin population in Pakistan has already experienced a 90% population decline due to illegal killing for scales and cultural beliefs^29,33,38^. Loss of suitable habitat under climate change could further impact pangolin distribution and abundance in the study area. The gain in suitable habitat of Indian pangolins was less than that of the overall predicted loss. However, 64.6–83.9% of currently suitable habitat may remain stable under changing climatic conditions. 

## Conclusion

Our findings predicted loss in suitable habitat of Indian pangolins ranged from 9.8 to 27.0% with the greatest loss observed under SSP 585 followed by SSP 126 (23.7%) during 2061–2080. The gain in suitable habitat of Indian pangolins was less than losses on average which ranged from 1.9 to 13.1% under all SSPs during all time periods. Most loss in suitable habitat was in the western and central parts while the least loss was in the eastern part of the study area. Under all SSPs, during 2041–2060 the gain in habitat was mostly in central parts while under all SSPs of 2061–2080, the gain in habitat was mostly in the western part of the study area. The eastern parts of the study areas were least impacted where most of the highly suitable habitat of Indian pangolin was distributed during current and future climatic scenarios. Our study provides insights into current and future suitable habitat of Indian pangolin which can help policy makers to identify priority areas for pangolin conservation in Pakistan in the face of climate change. Establishing new protected areas in areas of future suitable habitat and establishing conservation strategies could improve pangolin conservation now and in the future.

## Materials and methods

### Study area

Azad Jammu and Kashmir, Pakistan is located at 33° 50′ 36″ N, 73° 51′ 05″ E and includes 11 districts comprising 13,297 km^2^ (Fig. 1). Habitats range from tropical thorn forests to alpine scrub pastures with associated fauna and flora. Elevations are 223–5846 m above sea level. There are 22 protected areas comprising 1239.63 km^2^ (range 2–528 km^2^) in area. The annual mean temperature of the study area ranges between − 14 and 23 degrees centigrade while minimum and maximum temperature ranges between − 27.5 and 48 degrees centigrade^56^. Average annual rainfall ranges between 267 and 1375 mm^56^.

### Survey design

#### Survey design and occurrence data

Before starting the survey, approval was obtained from the Ethical Committee, Department of Wildlife Management, Pir Mehr Ali Shah, Arid Agriculture University, Rawalpindi (PMAS-AAUR/2646). All methods were carried out under relevant guidelines and regulations. Before interviewing human subjects, we obtained informed consent and informed respondents about the study objectives. We informally interviewed local people and staff of the AJ&K Wildlife & Fisheries Department to obtain information on Indian pangolin presence in the study area. Indian pangolins have been reported at elevations < 1540 m in AJ&K and Pakistan^29^ though our informal interviews suggested pangolins could occur at higher elevations. We, therefore, constrained our survey to areas < 2000 m. We divided the study area into a grid of 10- × 10-km cells and in each cell established 10 sampling points with a 500-m radius. We searched all accessible plots (topography, vegetation, and sites where we were able to reach) for direct (i.e., sighting) and indirect (burrows, fecal material, or tracks) signs of Indian pangolins during January 2021–June 2023. Locations of signs were recorded using a handheld GPS device. The indirect signs were confirmed by experts based on their shapes and sizes^29,45^. We also deployed cameras (UOVision UV557, Shenzen, China) at 90 sites to detect pangolins. The camera trap sites were selected based on the presence of active living burrows of Indian pangolins. We attached cameras to trees about 60 cm above ground for fifteen days at each site and programmed cameras to take three images for each detection^57^. We supplemented our data using location data of Indian pangolins previously collected in northern Pakistan^30–33,38,42,45,50^.

#### Bioclimatic data

We downloaded bioclimatic data for recent (1970–2000) and future (2041–2060, 2061–2080, 2081–2100) climate scenarios from WorldClim^56^ (https://www.worldclim.org/data/bioclim.html) and used the Hadley Global Environment Model (HadGEM3-GC31-LL) as it was best fit for our study area based on findings of previous studies^58–62^. We downloaded three Shared Socio-economic Pathways (SSPs): SSP 126, SSP 245, and SSP 585 for the years 2041–2060, 2061–2080, and 2081–2100. We masked all environmental layers to northern Pakistan and converted each to the same resolution (1 km^2^), projection, and American Standard Code for Information Interchange (ASCII). We assessed the pairwise correlation of variables using the autocorrelation feature in the Species Distribution Modeling toolbox (SDM) in ArcGIS (version 10.3). We assumed multicollinearity when |*r*| > 0.70^63,64^, and removed variables we considered less ecologically important. We used the spThin package^65^ in R software (version 4.2.2, R Development Core Team 2023) to rarify pangolin occurrence data to one occurrence per 1 km^2^ cell.

#### Species distribution model

We used MaxEnt software (version 3.4.1, http://biodiversityinformatics.amnh.org/open_source/maxent/)^66,67^ to model Indian pangolin distribution. Though there are reported limitations of MaxEnt^68–70^, there are also advantages such as few occurrence points are required to yield good results and reduced potential for model over-fitting^24,70–74^. We used default settings in MaxEnt but using logistic output format, 10,000 maximum number of iterations, LQHP feature types (occurrences > 80), with 10,000 background points and 10 replicates for our model. For regularization multiplier we used stepwise approach by successively running the model with different regularization multiplier values e.g., 0.5, 1.0, 1.5, 2.0, & 3.0 to constrain MaxEnt and avoid over-fitting of the model^66,70^. We used the jackknife test in Maxent^75^ to assess the contributions of bioclimatic predictors. We used the Area Under the Curve (AUC) and True Skill Statistics (TSS) to assess the predictive power of our models^76–79^. Values for AUC range from 0 to 1 with values > 0.9 considered excellent, > 0.8–0.9 very good, > 0.7–0.8 good, > 0.6–0.7 fair, and ≤ 0.6 poor^80^. We used the formula sensitivity + specificity − 1 to compute TSS, while sensitivity and specificity were calculated on the probability threshold for which their sum is maximized^79^. We then categorized predicted habitat into four classes: 0–0.1 (unsuitable), > 0.1–0.4 (less suitable habitat), > 0.4–0.7 (moderately suitable habitat), and > 0.7–1 (highly suitable habitat)^59,62,81,82^. We used the raster classify tool in Arc GIS (version 10.3, https://www.esri.com/arcgis-blog/products/3d-gis/3d-gis/arcgis-10-3-the-next-generation-of-gis-is-here/) to calculate the area of each habitat class during recent and future scenarios. Using classify raster and raster calculator tool we computed the amount of habitat lost, gained, and unchanged across periods for each category^83^.

## Supplementary Information

Supplementary Information.

## Data Availability

All data generated or analyzed during this study are included in this published article [Tables and Figures].
