# Correction: Predicting current and future habitat of Indian pangolin (*Manis crassicaudata*) under climate change

**DOI:** 10.1038/s41598-025-27761-9

**Published:** 2025-12-03

**Authors:** Siddiqa Qasim, Tariq Mahmood, Bushra Allah Rakha, Muhammad Sajid Nadeem, Faraz Akrim, Asad Aslam, Jerrold L. Belant

**Affiliations:** 1 Department of Zoology, Wildlife and Fisheries, PMAS Arid Agriculture University, Rawalpindi, Pakistan; 2 Department of Zoology, University of Kotli, Azad Jammu and Kashmir, Pakistan; 3 Department of Fisheries and Wildlife, Michigan State University, East Lansing, MI USA

Correction to: *Scientific Reports* 10.1038/s41598-024-58173-w, published online 30 March 2024

The original version of this Article contained a repeated error, where the bioclimatic variable Bio 12 was incorrectly defined as the "mean temperature of the coldest month" and "mean temperature of the coldest quarter". The correct definition is "annual precipitation."

As a result, in the Abstract,

“Model accuracy was very good (AUC = 0.885, TSS = 0.695), and jackknife tests of variable importance showed that the contribution of annual mean temperature (bio1) was greatest (33.4%), followed by the mean temperature of the coldest quarter (bio-12, 29.3%), temperature seasonality (bio 4, 25.9%), and precipitation seasonality (bio 15, 11.5%).”

now reads:

“Model accuracy was very good (AUC = 0.885, TSS = 0.695), and jackknife tests of variable importance showed that the contribution of annual mean temperature (bio12) was greatest (33.4%), followed by the annual precipitation (bio-12, 29.3%), temperature seasonality (bio 4, 25.9%), and precipitation seasonality (bio 15, 11.5%).”

In the Results,

“The habitat suitability for Indian pangolin increased with increasing mean temperature of the coldest month (bio12) and peaked (0.98) at 1100–1200 values.”

now reads:

“The habitat suitability for Indian pangolin increased with increasing annual precipitation (bio12) and peaked (0.98) at 1100–1200 values.”

In the Discussion,

“Our results demonstrated that four bioclimatic variables including annual mean temperature (bio1), mean temperature of coldest quarter (bio12), temperature seasonality (bio4), and precipitation seasonality (bio15) were most important in describing Indian pangolin occurrence. Among these, annual mean temperature (bio1) had the greatest contribution in describing the distribution of Indian pangolin, followed by mean temperature of the coldest quarter, temperature seasonality, and precipitation seasonality contributed the least to the maxent model.”

now reads:

“Our results demonstrated that four bioclimatic variables including annual mean temperature (bio1), annual precipitation (Bio 12), temperature seasonality (bio4), and precipitation seasonality (bio15) were most important in describing Indian pangolin occurrence. Among these, annual mean temperature (bio1) had the greatest contribution in describing the distribution of Indian pangolin, followed by annual precipitation (Bio 12), temperature seasonality, and precipitation seasonality contributed the least to the maxent model.”

Finally, the legend of Figure 3,

“Response of bioclimatic variable to habitat suitability of Indian pangolin (red line represents standard deviations, blue line represents effects of bioclimatic variables on predicted habitat suitability), northern Pakistan, 2021–2023. (A) Annual Mean Temperature (Bio1), (B) Temperature Seasonality (Bio 04), (C) Mean Temperature of Coldest Quarter (Bio12), D) Precipitation Seasonality (Bio 15).”

now reads:

“Response of bioclimatic variable to habitat suitability of Indian pangolin (red line represents standard deviations, blue line represents effects of bioclimatic variables on predicted habitat suitability), northern Pakistan, 2021–2023. (A) Annual Mean Temperature (Bio1), (B) Temperature Seasonality (Bio 04), (C) annual precipitation (Bio12), D) Precipitation Seasonality (Bio 15).”

The original Article has been corrected.

