# Supplementary material for: Predicting current and future habitat of Indian pangolin (Manis crassicaudata) under climate change
Source: Sci Rep. 2024 Mar 30;14:7564. doi: 10.1038/s41598-024-58173-w (PMC10981748; doi:10.1038/s41598-024-58173-w)
Supplement: Supplementary file 1 — Supplementary Information. [file 41598_2024_58173_MOESM1_ESM.pdf]

Predicting current and future habitat of Indian pangolin (*Manis crassicaudata*) under climate change

Authors

Siddiqa Qasim[1], Tariq Mahmood[1], Bushra Allah Rakha[1], Muhammad Sajid Nadeem[1], Faraz Akrim[2], Asad Aslam[2], Jerrold L. Belant[3]

[1]Department of Zoology, Wildlife and Fisheries, PMAS Arid Agriculture University, Rawalpindi, Pakistan

[2]Department of Zoology, University of Kotli, Azad Jammu and Kashmir, Pakistan

[3]Department of Fisheries and Wildlife, Michigan State University, East Lansing, Michigan, USA

Supplementary Material

Script of R software spThin Package used to rarify occurrence data of Indian pangolin

```
#rarefy occurrence data
library(spThin)
read.delim("data.txt", header = TRUE)# to read the data directly from working directory
data(data)
head( data )
table( data$REGION )
#Run spThin::thin on the full dataset
thinned_dataset_full <-
  thin( loc.data = data,
        lat.col = "LAT", long.col = "LONG",
        spec.col = "SPEC",
        thin.par = 1, reps = 5,
        locs.thinned.list.return = TRUE,
        write.files = FALSE,
```

```
        write.log.file = FALSE)
#Below is the same call, but in this case we are writing a number of
#files to disk. This files include a set of *.csv files of the thinned
#data and a log file.
thinned_dataset_full <-
  thin( loc.data = data,
        lat.col = "LAT", long.col = "LONG",
        spec.col = "SPEC",
        thin.par = 1, reps = 100,
        locs.thinned.list.return = TRUE,
        write.files = TRUE,
        max.files = 5,
        out.dir = "pangolin_thinned_full/", out.base = "pangolin_thinned",
        write.log.file = TRUE,
        log.file = "pangolin_thinned_full_log_file.txt" )
getwd()
```

#In the case above, we found that 10 repetitions were sufficient to return spatially thinned datasets with the optimal number of occurrence records (124). Because this is a random process, it is possible that a similarly repeated run would not return any datasets with the optimal number of occurrence records. To visually assess whether we are using enough reps to approach the optimal number we use the function plotThin, This function produces three plots: 1) the cumulative number of records retained versus the number of repetitions, 2) the log cumulative number of records retained versus the log number of repetitions, and 3) a histogram of the maximum number of records retained for each thinned dataset.

```
plotThin( thinned_dataset_full )
```
